# Prevalence and genetic diversity of *Echinorhynchus gymnocyprii* (Acanthocephala: Echinorhynchidae) in schizothoracine fishes (Cyprinidae: Schizothoracinae) in Qinghai-Tibetan Plateau, China

**DOI:** 10.1186/s13071-020-04224-w

**Published:** 2020-07-20

**Authors:** Meng-Tong Lei, Jin-Zhong Cai, Chun-Hua Li, Yong Fu, Jian Sun, Dou-Dou Ma, Yao-Peng Li, Yan-Ming Zhang

**Affiliations:** 1 College of Veterinary Medicine, Northwest A&F University, Yangling, 712100 Shaanxi People’s Republic of China; 2 Qinghai Academy of Animal Sciences and Veterinary Medicine, Qinghai University, State Key Laboratory of Plateau Ecology and Agriculture, Xining, 810016 Qinghai People’s Republic of China; 3 Qinghai Provincial Fishery Environmental Monitoring Center, Xining, 810012 Qinghai People’s Republic of China

**Keywords:** *Echinorhynchus gymnocyprii*, Schizothoracine fishes, Molecular characterization, Molecular phylogeny, Qinghai-Tibetan Plateau

## Abstract

**Background:**

The schizothoracine fishes, an excellent model for several studies, is a dominant fish group of the Qinghai-Tibet Plateau (QTP). However, species populations have rapidly declined due to various factors, and infection with *Echinorhynchus gymnocyprii* is cited as a possible factor. In the present study, the molecular characteristics of *E. gymnocyprii* in four species of schizothoracine fishes from the QTP were explored.

**Methods:**

We investigated the infection status of *E. gymnocyprii* in 156 schizothoracine fishes from the upper Yangtze River, upper Yellow River, and Qinghai Lake in Qinghai Province, China. The complete internal transcribed spacer (ITS) of the ribosomal RNA (rRNA) gene and part of the mitochondrial cytochrome *c* oxidase subunit 1 (*cox*1) gene of 35 *E. gymnocyprii* isolates from these fishes were sequenced and their characteristics analyzed. In addition, we inferred phylogenetic relationships of the *E. gymnocyprii* populations based on the rRNA-ITS and *cox*1 sequences.

**Results:**

The total prevalence of *E. gymnocyprii* in schizothoracine fishes was 57.69% (90/156). However, the prevalence among different species as well as that across the geographical locations of the schizothoracine fishes was significantly different. The results of sequence analysis showed that the four *E. gymnocyprii* populations from different hosts and regions of Qinghai Province were conspecific, exhibiting rich genetic diversity. Phylogenetic analysis based on rRNA-ITS and *cox*1 sequences supported the coalescence of branches within *E. gymnocyprii*; the *cox*1 gene of *E. gymnocyprii* populations inferred some geographical associations with water systems. In addition, three species of schizothoracine fishes were recorded as new definitive hosts for *E. gymnocyprii*.

**Conclusions:**

To the best of our knowledge, this is the first molecular description of *E. gymnocyprii* populations in schizothoracine fishes from the Qinghai-Tibet Plateau that provides basic data for epidemiological surveillance and control of acanthocephaliasis to protect endemic fish stocks.
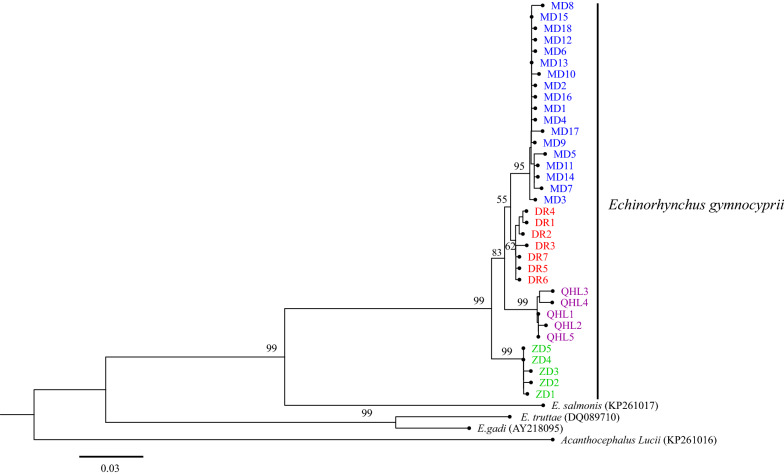

## Background

Acanthocephalans are a group of widely distributed intestinal helminths, commonly found in fishes worldwide; they can cause malnutrition, tissue injury, and intestinal obstruction in hosts [1–3]. *Echinorhynchus* Zoega in Müller, 1776 (Acanthocephala: Echinorhynchidae) is a group of spiny-headed worms that is mainly parasitic in teleost fish and crustaceans found in various aquatic environments. It is the most widely distributed genus of acanthocephalans [4]; 52 species of *Echinorhynchus* have been recorded [5]. Because of its high morphological variability, there is a debate on the subdivision of this genus, and morphological identification of the species of *Echinorhynchus* is difficult [4, 6]. Therefore, DNA sequence data from molecular markers have been used for classification, evolution, and phylogeny of the *Echinorhynchus* spp. [4, 7, 8]. *Echinorhynchus gymnocyprii* Liu, Wang & Yang, 1981, with an elongated and cylindrical proboscis, and 14–16 longitudinal rows of hooks (each row having 10–11 hooks of unequal length), was first described in *Gymnocypris przewalskii* (Kessler, 1876) in the Qinghai Lake of China [9]. Later, its presence was discovered in *Triplophysa scleroptera* (Herzenstein, 1888) in the Qinghai Lake and in *T. stenura* (Herzenstein, 1888) in the Lhasa River in Tibet [10, 11]. However, to date, there has been no molecular data on *E. gymnocyprii*.

The schizothoracine fishes (Teleostei: Cyprinidae) are a dominant fish group of the Qinghai Tibet Plateau (QTP) ichthyofauna, and China has the largest distribution of schizothoracine species worldwide [12–14]. These fishes are endemic and adapted to the extreme environmental conditions (e.g. hypoxia, strong ultraviolet rays, food shortages and cold) of the QTP [12, 15–17]. Therefore, they are ideal models to investigate speciation, biological evolution, ecological effects, paleo-drainage changes, and biogeography of the QTP [13, 17–20]. However, a variety of factors have led to a sharp decline in the population of schizothoracine fishes in the last decades [21, 22]. Parasites found in these fishes may contribute to this decline, as some studies suggest that parasites may have adverse effects on their host populations [23, 24]. As an important part of QTP biodiversity, parasites of endemic fishes have gradually attracted the attention of researchers [11, 25].

During the investigation of the helminth fauna of schizothoracine fishes in the QTP of China, it was found that these fishes were infected with acanthocephalans, which were initially identified as *E. gymnocyprii* based on morphological studies. In the present study, the infection status of *E. gymnocyprii* in four species of schizothoracine fishes from the upper Yangtze River, Qinghai Lake, and upper Yellow River in the QTP was evaluated. The molecular data on *E. gymnocyprii* populations from different hosts and geographical locations (including the *E. gymnocyprii* population from the type-host, *G. przewalskii*, and type-locality of Qinghai Lake) are described based on ribosomal RNA (rRNA), which includes the complete internal transcribed spacer (ITS) and the mitochondrial cytochrome *c* oxidase subunit 1 (*cox*1) gene. In addition, the phylogenetic relationships of the *E. gymnocyprii* populations were investigated based on the rRNA-ITS and *cox*1 sequences.

## Methods

### Sampling

**Fig. 1:**
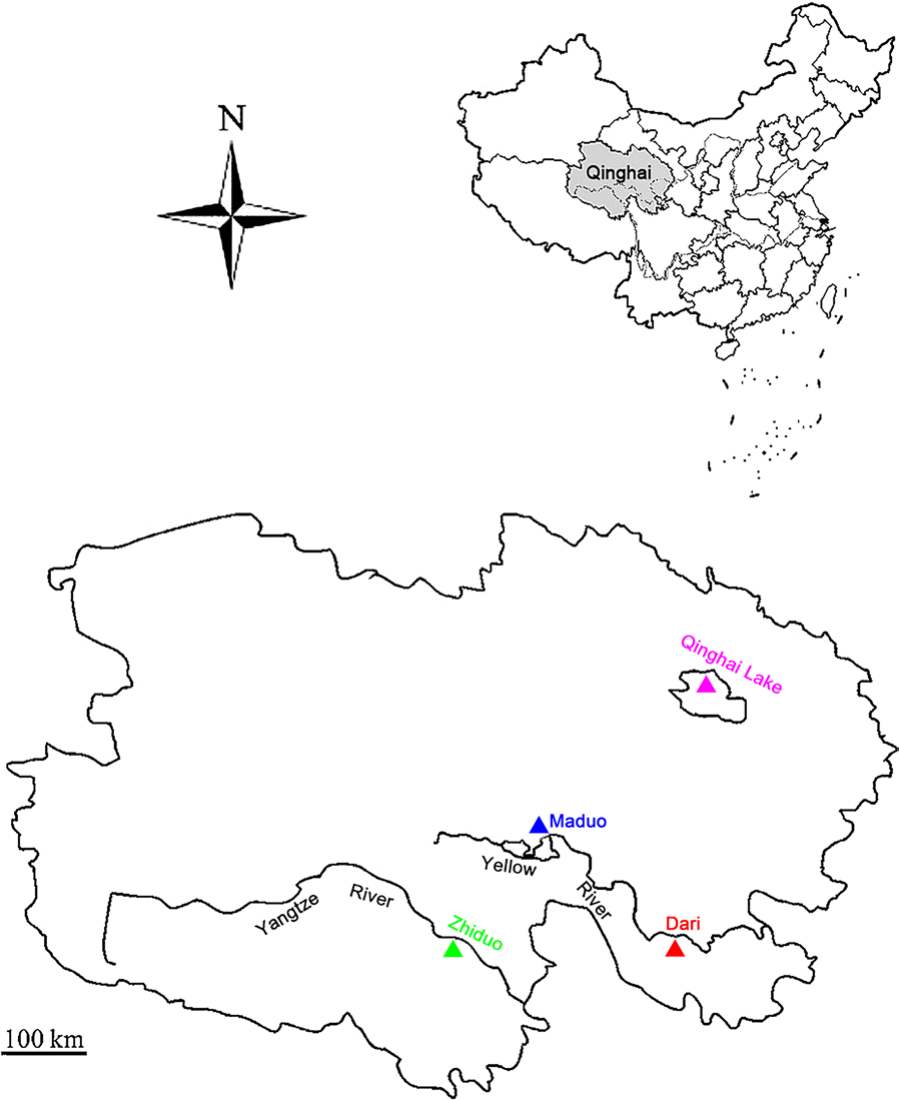
Collection sites of *E. gymnocyprii* in the Qinghai Province of China

A total of 156 schizothoracine fishes (20 *Ptychobarbus kaznakovi* Nikolskii, 1903, 73 *Gymnocypris eckloni* Herzenstein, 1891, 11 *Gymnodiptychus pachycheilus* Herzenstein, 1892, and 52 *Gymnocypris przewalskii*), from four localities and three water systems in the northeastern QTP of Qinghai Province, China, were examined for parasites (Fig. 1). The sampling locations were in Zhiduo County (ZD) of the upper Yangtze River, Maduo County (MD) and Dari County (DR) of the upper Yellow River, and Qinghai Lake (QHL); the altitudes of ZD, MD, DR, and QHL are 4285 m, 4300 m, 3970 m and 3196 m, respectively. The Yangtze and Yellow rivers are both freshwater rivers, and the Qinghai Lake is a closed-basin brackish lake. The acanthocephalans were washed immediately in saline after collection from the intestines of fishes, preserved in 70% ethanol, and stored at 4 °C. The preliminary identification of *E. gymnocyprii* was mainly based on morphological characteristics (elongated and cylindrical proboscis, armed with 14–16 longitudinal rows of hooks, each row having 10–11 hooks of unequal length) [9, 26]. Subsequently, 35 acanthocephalans were selected for DNA analysis. The *E. gymnocyprii* found in *G. przewalskii* of Qinghai Lake was the type-species.

### DNA extraction, amplification, cloning and sequencing

Before performing genomic DNA analysis, the anterior extremities of the acanthocephalans were cut off to avoid contaminating the DNA with fish tissues attached to the proboscis. Next, the genomic DNA of individual acanthocephalans was extracted using the QIAamp® DNA Mini kit (Qiagen, Hilden, Germany) according to the manufacturer’s recommendations.

We used the primers 18SF1 (5′-GCG AAG CAT TTG CCA AGA A-3′) and NC2 (5′-TTA GTT TCT TTT CCT CCG CT-3′) to amplify the fragment of rRNA-ITS (including partial *18S* rRNA gene, ITS1; *5.8S* rRNA gene, ITS2; and partial *28S* rRNA gene) [7]. A partial DNA fragment of the *cox*1 gene was also amplified using the forward primer (5′-AGT TCT AAT CAT AA(R) GAT AT(Y) GG-3′) and the reverse primer (5′-TAA ACT TCA GGG TGA CCA AAA AAT CA-3′) [27].

Each PCR mixture (50 μl) consisted of 25 μl 2× pfu PCR MasterMix (Zomanbio, Beijing, China), 0.5 μl of each primer (0.5 μM), 23 μl ddH2O, and 1 μl genomic DNA. The PCR conditions for each gene were as follows: rRNA-ITS [7]: 93 °C for 3 min (pre-denaturation), followed by 35 cycles at 93 °C for 1 min (denaturation), 48 °C for 1 min (annealing), 72 °C for 2 min (extension), followed by a final extension step at 72 °C for 3 min; *cox*1 gene: 95 °C for 5 min (pre-denaturation), 94 °C for 30 s (denaturation), 52 °C for 30 s (annealing), 72 °C for 1 min (extension), each for 35 cycles, and a final extension step at 72 °C for 10 min. All amplicons were tested by electrophoresis on a 1% agarose gel with ExRed (Zomanbio). The PCR products were purified using a DNA Gel Extraction Kit (Axygen, Union, USA), cloned with a pMD™19-T vector, and then transformed in DH5α *Escherichia coli* cells (TaKaRa, Dalian, China). The positive plasmid DNA samples were sent to Sangon Company (Shanghai, China) for sequencing.

### Sequence analysis

The sequence obtained from each acanthocephalan was assembled using DNAStar software [28] and then aligned with Clustul X 2.0 [29] and edited manually. Subsequently, the data obtained from these corrected sequences were analyzed with MEGA 6.06; both the pairwise distance and the mean distance within/between the populations were estimated using the Kimura 2-parameter model [30, 31]. In addition, the sequences were compared with sequences from GenBank by BLAST analysis. The sequences of ITS1 and ITS2 were defined in a previous study [32]. Based on the ITS1, ITS2 and *cox*1 sequences of each population, haplotype analysis was carried out using DNASP 5.10 [33].

### Phylogenetic analysis

The rRNA-ITS and *cox*1 sequences were independently analyzed and aligned with sequences from GenBank. The rRNA-ITS sequences were as follows: *E. gadi* Zoega in Müller, 1776 (EF107647, EF107648), *Pomphorhynchus laevis* Zoega in Müller, 1776 (KJ756500), *P. bosniacus* Kistaroly & Cankovic, 1969 (MH319900), and *P. zhoushanensis* (KY472823); the *cox*1 sequences were: *E. gadi* (AY218095), *E. salmonis* Müller, 1784 (KP261017), *E. truttae* Schrank, 1788 (DQ089710), and *Acanthocephalus lucii* Müller, 1776 (KP261016), according to the software Clustal X 2.0 [29]. After amendment, the rRNA-ITS and *cox*1 datasets included 633 characters and 585 characters, respectively. Phylogenetic trees were constructed by the neighbor-joining (NJ) method using MEGA 6.06 [30] and Maximum Likelihood (ML) with IQ tree 1.6.10 [34]. For NJ, the Kimura 2-parameter model was used. The TPM3 + F + I for the rRNA-ITS dataset, and the HKY + F + I for the *cox*1 dataset, were suggested as the best-fit models by ModelFinder [35] of IQ tree 1.6.10 based on the Bayesian information criterion (BIC). *Pomphorhynchus zhoushanensis* and *A. lucii* were selected as the outgroups of the rRNA-ITS and *cox*1 datasets, respectively. The branch reliability of the phylogenetic tree constructed by the two methods was tested by bootstrapping with 1000 replicates. A haplotype network was constructed from the *cox*1 DNA sequence data with the software TCS using the probabilistic method of statistical parsimony [36, 37].

### Statistical analysis

The differences in *E. gymnocyprii* infection rates among different fishes and locations were analyzed using the chi-square test in SPSS 25.0 for Windows (IBM Corp., New York, USA). *P* < 0.05 was considered as statistically significant.

## Results

### Prevalence of *E. gymnocyprii* in schizothoracine fishes

The morphological characters of *E. gymnocyprii* based on samples collected from different hosts and locations were similar, with a minor variation in size (Additional file 1: Table S1). *Echinorhynchus gymnocyprii* was found in 90 of 156 (57.69%) fish samples, with infection rates ranging from 45% (*G. pachycheilus*) to 100% (*P. kaznakovi*) among the four species of schizothoracine fishes (*χ*^2^ = 15.919, *df* = 3, *P* = 0.0012), The prevalence of these helminths varied across different geographical localities; among the four locations in Qinghai Province, the prevalence ranged from 40.00% in Dari to 100.00% in Maduo (*χ*^2^ = 26.425, *df* = 3, *P* < 0.0001) (Table 1). In addition, the mean intensity of *E. gymnocyprii* infection ranged from 14.9 (*G. eckloni* in Dari) to 41.4 (*G. pachycheilus* in Maduo) (Table 1).

**Table 1:** Occurrence of *E. gymnocyprii* in schizothoracine fishes in the Qinghai Province

Location	*n*	*Ptychobarbus kaznakovi*		*Gymnocypris eckloni*		*Gymnodiptychus pachycheilus*			
		P (%)	MI	P (%)	MI	P (%)	MI	P (%)	MI
Zhiduo	20	45.00 (9/20)	19.4 (5–27)	–	–	–	–	–	–
Maduo	19	–	–	100.00 (14/14)	34.3 (2–234)	100.00 (5/5)	41.4 (9–95)	–	–
Dari	65	–	–	33.90 (20/59)	14.9 (1–98)	100.00 (6/6)	16.3 (8–41)	–	–
Qinghai Lake	52	–	–	–	–	–	–	69.23 (36/52)	24.0 (1–98)
Total	156	45.00	19.4	46.58	22.9	100.00	27.7	69.23	24.0

*Abbreviations*: *n*, number of fish samples; P, prevalence (number of positive/total number examined); MI, mean intensity

### Sequence analysis

The locations, sample numbers, sex, and hosts of *E. gymnocyprii* are shown in Table 2. The rRNA-ITS region was 1493-1497 bp in length (259–263 bp for ITS1, 85 bp for *5.8S* and 251 bp for ITS2 rDNA) (GenBank: MT162052–MT162085). The percentages of nucleotide identity and variations in the number of nucleotides within *E. gymnocyprii* populations, based on ITS1 (259–263 bp) and ITS2 (251 bp), are shown in Additional file 2: Table S2 and Additional file 3: Table S3, respectively. The ITS1 of *E. gymnocyprii* from QHL4-5, MD1-3, MD7-13, MD15-17 and DR2-5 were 100% identical; ZD1, ZD3 and ZD5 were 100% identical (where QHL: Qinghai Lake; MD: Maduo; DR: Dari; and ZD: Zhiduo), and the percent identity within *E. gymnocyprii* was 96.9–100.0%. The ITS2 of QHL1, QHL3-5, MD2, MD4-7, MD9-13, MD15-18, DR2-4 and ZD1-5 were 100% identical, despite being found at different locations (QHL, MD, DR and ZD) and in different hosts (*G. przewalskii*, *G. eckloni*, *Gymnodiptychus pachycheilus* and *P. kaznakovi*); the percent identity within *E. gymnocyprii* was 98.4–100%. The percent identity based on the ITS1 and ITS2 between *E. gymnocyprii* and *E. gadi* (GenBank: EF107647) was 54.0–56.2% (Additional file 2: Table S2, Additional file 3: Table S3).

**Table 2:** *Echinorhynchus gymnocyprii* samples used in the present study

Host	Collection site	Code	Sex	GenBank ID	
				rRNA	*cox*1
*Gymnocypris eckloni*	Maduo	MD1	Female	MT162052	MT169741
	Maduo	MD2-8	Male	MT162053–MT162059	MT169742–MT169748
	Maduo	MD14-18	Female	MT162065–MT162069	MT169754–MT169758
	Dari	DR1	Female	–	MT169759
	Dari	DR2, 3	Female	MT162070, MT162071	MT169760, MT169761
	Dari	DR6-7	Male	MT162074, MT162075	MT169764, MT169765
*Ptychobarbus kaznakovi*	Zhiduo	ZD1, 3	Male	MT162081, MT162083	MT169771, MT169773
	Zhiduo	ZD2	Male	MT162082	MT169772
	Zhiduo	ZD4-5	Female	MT162083–MT162085	MT169774, MT169775
*Gymnodiptychus pachycheilus*	Maduo	MD9-10	Male	MT162060, MT162061	MT169749, MT169750
	Maduo	MD11-13	Female	MT162062–MT162064	MT169751–MT169753
	Dari	DR4	Female	MT162072	MT169762
	Dari	DR5	Male	MT162073	MT169763
*Gymnocypris przewalskii*	Qinghai Lake	QHL1-3	Male	MT162076–MT162078	MT169766–MT169768
	Qinghai Lake	QHL4-5	Female	MT162079, MT162080	MT169769, MT169770

Regarding the *cox*1 gene, 35 newly generated sequences were 702 bp long; the sequences of QH1 and QH5 were identical, and the sequences of ZD4 and ZD5 were identical. The overall *cox*1 GC content was 37.3%, showing a high AT bias; the percent identity between these sequences was 95.4–100.0%. The overall average of pairwise distances was 0.02182, and the percent identity/mean distance within each population was 98.8–100.0%/0.00599 for MD, 99.0–99.7%/0.00697 for DR, 98.8–100.0%/0.00862 for QHL, and 99.3–100.0%/0.00401 of ZD, respectively. The percent identity/mean distance of the QHL population and MD, DR and ZD populations was 95.9–97.3%/0.0344, 96.8–98.1%/0.0236 and 95.4–96.6%/0.0385, respectively. There are six other *Echinorhynchus* species with *cox*1 gene sequences deposited on GenBank, and the mean pairwise comparison between the *E. gymnocyprii* population in our study and these species yielded 0.2386 (*E. salmonis*, GenBank: KP261017) to 0.4263 (*E. cinctulus*, GenBank: KP261014) nucleotide differences. The *cox*1 sequences of *E. gymnocyprii* have been deposited in GenBank under the accession numbers MT169741–MT169775. The summary statistics for the *cox*1 gene in *E. gymnocyprii* populations are shown in Table 3, and the values of h, k, and π indicate that the populations of *E. gymnocyprii* are rich in genetic diversity.

**Table 3:** Summary statistics observed in *E. gymnocyprii* populations based on *cox*1 gene sequences

Population code	*n*	h	C	V	Pi	S	Hd	π	k
MD	18	18	688	14	4	10	1.000	0.00592	4.17647
DR	7	7	670	32	3	29	1.000	0.00689	4.85714
QHL	5	4	695	7	-	7	0.900	0.00851	6.00000
ZD	5	4	688	14	2	12	0.900	0.00397	2.80000
Total	35	33	619	83	36	47	0.997	0.02122	14.95966

*Abbreviations*: *n*, number of sequenced individuals; h, number of haplotypes; C, conserved sites; V, variable sites; S, singleton sites; Pi, parsimony-informative sites; Hd, Haplotype diversity; *π*, nucleotide diversity; S, number of segregating sites; k, average number of nucleotide differences

### Phylogenetic analyses

**Fig. 2:**
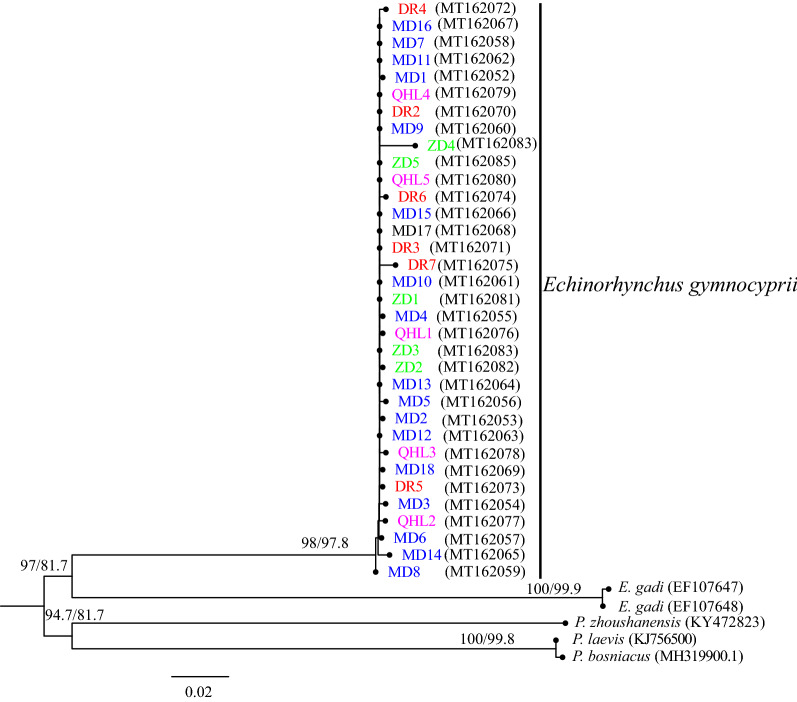
Phylogenetic relationships of *E. gymnocyprii*. The tree is inferred from the rRNA-ITS sequences using neighbor-joining (NJ) method. The maximum likelihood (ML) method produced phylogenetic tree with the same branch topologies. Bootstrap support from ML/NJ analysis are shown above the nodes. The scale-bar indicates the number of substitutions per site. *Pomphorhynchus zhoushanensis* was used as the outgroup

**Fig. 3:**
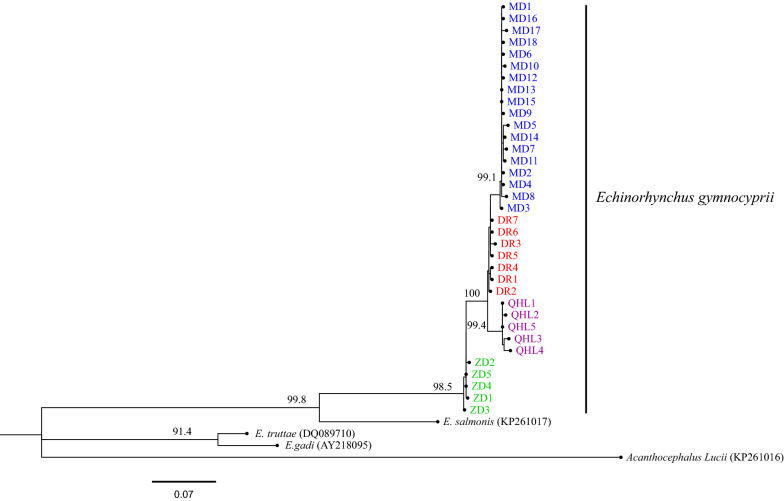
Phylogenic tree based on *cox*1 gene sequences of *E. gymnocyprii* using the maximum likelihood (ML) method. Bootstrap support is shown above the nodes. The scale-bar indicates the number of substitutions per site. Branch labels of different colors indicate different locations of *E. gymnocyprii* populations in this study (blue, Maduo County (MD); red, Dari County (DR); purple, Qinghai Lake (QHL); green, Zhiduo County (ZD).) *Acanthocephalus lucii* was used as the outgroup. The branches of the phylogram depicting the relationships among the *E. gymnocyprii* sequences are shown in Additional file 4: Figure S1

**Fig. 4:**
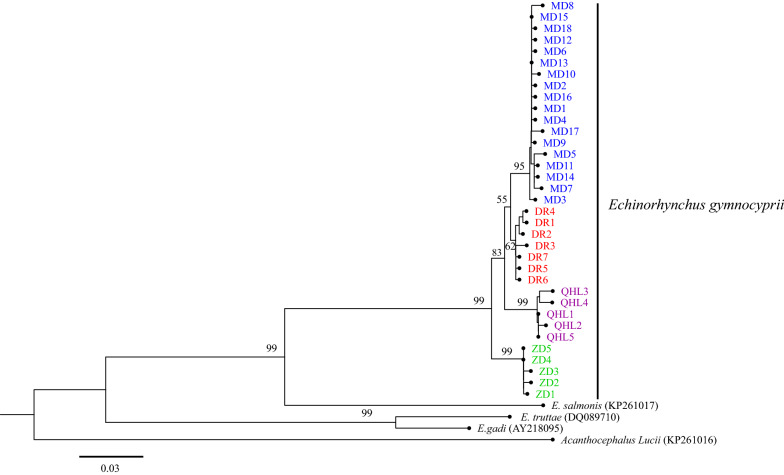
Phylogenic tree based on *cox*1 gene sequences of *E. gymnocyprii* using the Neighbor-Joining (NJ) method. Bootstrap support is shown above the nodes. The scale-bar indicates genetic distance. Branch labels of different colors indicate different sources of *E. gymnocyprii* populations in this study (blue, Maduo County (MD); red, Dari County (DR); purple, Qinghai Lake (QHL); green, Zhiduo County (ZD)). *Acanthocephalus lucii* was used as the outgroup

The topological structures of the phylogenetic trees based on the rRNA-ITS and *cox*1 sequences of the *E. gymnocyprii* populations were similar; all the *E. gymnocyprii* populations clustered in a single well-supported clade, with only slight differences in the bootstrap values for some nodes (Figs. 2, 3, 4). The phylogenetic NJ tree of *E. gymnocyprii* populations based on the *cox*1 sequences, inferred 4 subclades (subclades MD, DR, QHL and ZD; Fig. 4), corresponding to 4 geographical locations belonging to 3 water systems. The MD and DR groups belong to the Yellow River system, the QHL group belongs to the Qinghai Lake system, and the ZD group belongs to the Yangtze River system. However, the DR and ZD groups appear to be paraphyletic in the ML tree based on the *cox*1 sequences (Fig. 3).

**Fig. 5:**
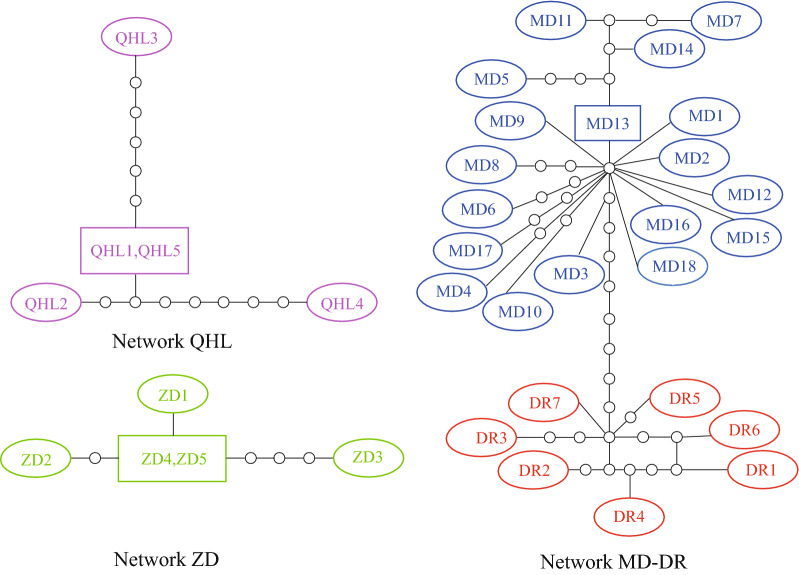
Statistical parsimony network of *E. gymnocyprii* haplotype based on *cox*1 gene sequences. The connection limit excluding homoplasic changes was set to 95%. Each oval represents a haplotype, and the ancestral haplotype (with the highest outgroup probability) is indicated by a square, the size of the square or oval corresponds the haplotype frequency. Each line equates to one mutational step, and the small circles are hypothetical haplotypes

The 35 specimens comprised 33 unique haplotypes, and the statistical parsimony analysis revealed 3 distinct networks (QHL, ZD and MD-DR; Fig 5), corresponding to three water systems. Network QHL contained 4 haplotypes from 5 individuals, QHL1 and QHL5 were considered the central haplotype; network ZD contained 4 haplotypes from 5 individuals, ZD4 and ZD5 were considered the central haplotype; network MD-DR contained 25 haplotypes from 25 individuals, with MD13 as the ancestral haplotype.

## Discussion

In this study, we found that the prevalence and intensity of *E. gymnocyprii* from *G. przewalskii* in Qinghai Lake had similarity to those reported in previous studies [10]. Previously, a range of 33.9–100% for prevalence and 14.9–41.4 for mean intensity of *E. gymnocyprii* in *G. eckloni*, *Gymnodiptychus pachycheilus*, and *P. kaznakovi* was reported. In addition, the prevalence of *E. gymnocyprii* in schizothoracine fishes differed significantly depending on fish species and location. There are several possible explanations for these differences, such as the characteristics (e.g. feeding behavior, age, physiology and abundance) of different definitive hosts, different numbers of samples, and the ecological environment (e.g. density of intermediate hosts, aquatic organisms, water conditions and altitude) [38–44]; these differences result in different survival conditions for both the schizothoracine fishes and acanthocephalans. Our results suggest that *E. gymnocyprii* is widely distributed in native fishes of the QTP, a finding that should be considered when working to protect the schizothoracine fish population. Further studies of *E. gymnocyprii* may serve as a model for studying the co-evolution of native fishes and parasites in the QTP.

However, the host species, geographical distribution, age, and other factors may cause changes in the morphology of *Echinorhynchus* spp. These changes sometimes exceed the suggested generic boundaries, thereby leading to inconvenience and confusion in the classification and identification of *Echinorhynchus* spp. [2, 4, 6, 45–47]. Therefore, DNA sequences from the ribosomal gene cluster (e.g. *18S*, ITS and *28S*) or mitochondria (e.g. *cox*1) provide a complementary methodology for analyzing the taxonomy and phylogeny of *Echinorhynchus* spp. [4, 7, 47]. Taken together, these results support the use of rRNA (containing ITS) and the *cox*1 gene as genetic markers for the identification of *E. gymnocyprii*.

In our study, the values of π, k, and Hd based on the *cox*1 gene indicated that the *E. gymnocyprii* populations exhibit rich genetic diversity [33], which may explain the existence of these populations in a variety of endemic fish in the QTP and their adaptation to the harsh environment of the plateau. The overall π (0.02122) and k (14.95966) were significantly higher than those of π (0.00397–0.00851) and k (2.800–6.000) of single populations from MD, DR, QHL and ZD, which indicates that geographical factors may have a positive impact on the evolution of the *cox*1 gene in the *E. gymnocyprii* populations.

The phylogenetic analysis based on the rRNA-ITS and *cox*1 gene supports the coalescence of branches within the *E. gymnocyprii* populations, and provides evidence that these populations belong to the same species. Interestingly, the NJ tree based on the *cox*1 gene showed four subclades (Fig. 4), and the statistical parsimony analysis revealed three distinct networks (Fig. 5), both reflecting geographical associations with water systems. The *E. gymnocyprii* populations from the Yellow River system (MD, DR and QHL) were more closely related to each other than to those of the Yangtze River system (ZD). This result might be explained by geological research findings, which imply that the Qinghai Lake was connected to the ancient Yellow River approximately 0.15 mega-annum before the present (Ma BP) [48]. The distribution and evolution of schizothoracine fishes, as hosts of *E. gymnocyprii*, in the QTP are strictly due to differences in water systems and habitats [15, 49–52]. The *cox*1 gene of *E. gymnocyprii* populations showed some geographical associations with water systems, indicating that adaptive changes in the *cox*1 gene enabled the species to survive in different water environments and preserve genetic differentiation. Consequently, the *cox*1 gene is potentially useful for investigating the zoogeography of *E. gymnocyprii*.

In the present study, to the best of our knowledge, two DNA markers of *E. gymnocyprii* were analyzed for the first time, and three new host records were added, which may be the first step in further understanding the speciation and evolution of this species in the QTP. This study also provides important molecular data for revision of the genus *Echinorhynchus* [4] in the future.

## Conclusions

*Echinorhynchus gymnocyprii* is widely distributed in native fish of QTP; three species of schizothoracine fishes (*G. eckloni*, *Gymnodiptychus pachycheilus* and *P. kaznakovi*) were identified as new definitive hosts of *E. gymnocyprii*. Our study is the first molecular characterization of *E. gymnocyprii* populations in schizothoracine fishes from the QTP; it provides basic data for epidemiological surveillance and control of acanthocephaliasis to protect endemic fish stocks in the QTP.

## Supplementary information

**Additional file 1: Table S1.** Comparison of the specific characters of *E. gymnocyprii* from different hosts and locations.

**Additional file 2: Table S2.** Percentage nucleotide identity and numbers of nucleotide variations of ITS1 fragments from *E. gymnocyprii* populations.

**Additional file 3: Table S3.** Percentage nucleotide identity and numbers of nucleotide variations of ITS2 fragments from *E. gymnocyprii* populations.

**Additional file 4: Figure S1.** A phylogram form maximum likelihood (ML) analysis based on *cox*1 gene sequences depicting the relationships among the *E. gymnocyprii* sequences.

## Data Availability

The datasets supporting the findings of this article are included within the article and its additional files. The nuclear rDNA-ITS and *cox*1 nucleotide sequences generated in this study were deposited in the GenBank database under the accession numbers MT162052–MT162085 and MT169741–MT169775, respectively.

## Abbreviations

BIC: Bayesian information criterion
C: conserved sites
ITS: internal transcribed spacer
k: average number of nucleotide difference
Ma BP: mega-annum before present
ML: maximum likelihood
NJ: neighbor-joining
Pi: parsimony-informative sites
QTP: Qinghai-Tibetan plateau
S: singleton sites
V: variable sites
