# Supplementary material for: Prevalence and genetic diversity of Echinorhynchus gymnocyprii (Acanthocephala: Echinorhynchidae) in schizothoracine fishes (Cyprinidae: Schizothoracinae) in Qinghai-Tibetan Plateau, China
Source: Parasit Vectors. 2020 Jul 20;13:357. doi: 10.1186/s13071-020-04224-w (PMC7372853; doi:10.1186/s13071-020-04224-w)
Supplement: Supplementary file 1 — Additional file 1: Table S1. Comparison of the specific characters of E. gymnocyprii from different hosts and locations. [file 13071_2020_4224_MOESM1_ESM.docx]

**Additional file 1: Table S1** Comparison of the specific characters of *E. gymnocyprii* from different hosts and locations

| Host/ location | *G.Przewalskii*/ QHL | | *G. eckloni/*MD | | *G. eckloni/*DR | | *G.Pachycheilus/*MD | | *G.Pachycheilus/* DR | | *P.Kaznakovi/* ZD | |
| --- | --- | --- | --- | --- | --- | --- | --- | --- | --- | --- | --- | --- |
| Sex | Male | Female | Male | Female | Male | Female | Male | Female | Male | Female | Male | Female |
| Trunk shape | elongate, cylindrical | elongate, cylindrical | elongate, cylindrical | elongate, cylindrical | elongate, cylindrical | elongate, cylindrical | elongate, cylindrical | elongate, cylindrical | elongate, cylindrical | elongate, cylindrical | elongate, cylindrical | elongate, cylindrical |
| Trunk length (mm) | 6.2-9.4 | 15.7-21.6 | 7.5-9.9 | 14.6-19.2 | 6.9-8.4 | 11.3-18.9 | 5.9-8.3 | 11.9-19.1 | 5.7-8.6 | 13.7-20.8 | 7.5-9.4 | 17.9-21.0 |
| Trunk width (mm) | 0.9-1.4 | 1.1-1.8 | 0.87-1.1 | 1.0-1.4 | 0.7-1.1 | 0.9-1.3 | 0.8-1.3 | 0.9-1.7 | 0.7-1.0 | 1.0-1.6 | 0.8-1.1 | 1.1-1.6 |
| Proboscis shape | elongate, cylindrical | elongate, cylindrical | elongate, cylindrical | elongate, cylindrical | elongate, cylindrical | elongate, cylindrical | elongate, cylindrical | elongate, cylindrical | elongate, cylindrical | elongate, cylindrical | elongate, cylindrical | elongate, cylindrical |
| Proboscis length (μm) | 626.4- 1042.7 | 686.0- 1189.8 | 786.0-1156.1 | 749.9-1233.4 | 701.4-903.9 | 765.2-1250.1 | 908.7-1200.3 | 986.8-1137.5 | 744.8-857.0 | 707.8-967.2 | 796.1-910.3 | 806.5- 972.1 |
| Proboscis width (μm) | 294.3- 357.1 | 336.1- 448.0 | 347.6-475.6 | 404.4-504.6 | 287.1-352.7 | 310.1-401.2 | 275.2-387.6 | 402.2-508.2 | 264.6-372.7 | 367.3-419.9 | 167.5-312.4 | 393.5-452.8 |
| Number of rows of hooks | 14-16 | 14-16 | 14-16 | 14-16 | 14-16 | 14-16 | 14-16 | 14-16 | 14-16 | 14-16 | 14-16 | 14-16 |
| Number of hooks per row | 10-11 | 10-11 | 10-11 | 10-11 | 10-11 | 10-11 | 10-11 | 10-11 | 10-11 | 10-11 | 10-11 | 10-11 |

QHL Qinghai Lake, DR Dari, MD Maduo, ZD Zhiduo
