# Supplementary material for: Prevalence and genetic diversity of Echinorhynchus gymnocyprii (Acanthocephala: Echinorhynchidae) in schizothoracine fishes (Cyprinidae: Schizothoracinae) in Qinghai-Tibetan Plateau, China
Source: Parasit Vectors. 2020 Jul 20;13:357. doi: 10.1186/s13071-020-04224-w (PMC7372853; doi:10.1186/s13071-020-04224-w)
Supplement: Supplementary file 2 — Additional file 2: Table S2. Percentage nucleotide identity and numbers of nucleotide variations of ITS1 fragments from E. gymnocyprii populations. [file 13071_2020_4224_MOESM2_ESM.docx]

**Additional file 2: Table S2** Percentage nucleotide identity and numbers of nucleotide variations of ITS1 fragments from *E. gymnocyprii* populations

|  | QHL1 | QHL2 | QHL3 | QHL4 | MD4 | MD5 | MD6 | MD14 | MD18 | DR6 | DR7 | ZD1 | ZD2 | ZD4 | *E. gadi* |
| --- | --- | --- | --- | --- | --- | --- | --- | --- | --- | --- | --- | --- | --- | --- | --- |
| QHL1 | - | 99.2 | 99.2 | 99.6 | 99.2 | 99.2 | 98.8 | 97.7 | 99.2 | 99.2 | 98.4 | 99.2 | 98.8 | 98.8 | 54.8 |
| QHL2 | 2 | - | 99.2 | 99.6 | 99.2 | 99.2 | 98.8 | 97.7 | 99.2 | 99.2 | 98.4 | 99.2 | 98.8 | 98.8 | 55.5 |
| QHL3 | 2 | 2 | - | 99.6 | 99.2 | 99.2 | 98.8 | 97.7 | 99.2 | 99.2 | 98.8 | 99.2 | 98.8 | 98.8 | 55.1 |
| QHL4 | 1 | 1 | 1 | - | 99.6 | 99.6 | 99.2 | 98.0 | 99.6 | 99.6 | 98.8 | 99.6 | 99.2 | 99.2 | 55.1 |
| MD4 | 2 | 2 | 2 | 1 | - | 99.2 | 98.8 | 97.7 | 99.2 | 99.2 | 98.4 | 99.2 | 98.8 | 98.8 | 55.1 |
| MD5 | 2 | 2 | 2 | 1 | 2 | - | 98.8 | 97.7 | 99.2 | 99.2 | 98.4 | 99.2 | 98.8 | 98.8 | 55.1 |
| MD6 | 3 | 3 | 3 | 2 | 3 | 3 | - | 97.3 | 98.8 | 98.8 | 98.0 | 98.8 | 98.4 | 98.4 | 55.0 |
| MD14 | 6 | 6 | 6 | 5 | 6 | 6 | 7 | - | 97.7 | 97.7 | 96.9 | 97.7 | 97.3 | 97.3 | 54.0 |
| MD18 | 2 | 2 | 2 | 1 | 2 | 2 | 3 | 6 | - | 99.2 | 98.4 | 99.2 | 98.8 | 98.8 | 54.8 |
| DR6 | 2 | 2 | 2 | 1 | 2 | 2 | 3 | 6 | 2 | - | 98.4 | 99.2 | 98.8 | 98.8 | 55.1 |
| DR7 | 4 | 4 | 3 | 3 | 4 | 4 | 5 | 8 | 4 | 4 | - | 98.4 | 98.0 | 98.0 | 54.8 |
| ZD1 | 2 | 2 | 2 | 1 | 2 | 2 | 3 | 6 | 2 | 2 | 4 | - | 99.6 | 99.6 | 54.9 |
| ZD2 | 3 | 3 | 3 | 2 | 3 | 3 | 4 | 7 | 3 | 3 | 5 | 1 | - | 99.2 | 54.6 |
| ZD4 | 3 | 3 | 3 | 2 | 3 | 3 | 4 | 7 | 3 | 3 | 5 | 1 | 2 | - | 54.9 |
| *E.gadi* | 122 | 120 | 121 | 121 | 121 | 121 | 121 | 124 | 122 | 121 | 122 | 122 | 123 | 122 | - |

Percentage nucleotide identity (above the diagonal) , numbers of nucleotide variations (below the diagonal), ITS1 fragments of *E. gymnocyprii* populations (259-263 bp). The ITS1 fragments of QHL4-5, MD1-3, MD7-13, MD15-17, and DR2-5 were identical; therefore, data on only QHL4 is presented in this table; ZD1, ZD3, and ZD5 were identical, therefore, data on only ZD1 is shown in this table. *E. gadi*, (EF107647.1) 250 bp.
