# Supplementary material for: Prevalence and genetic diversity of Echinorhynchus gymnocyprii (Acanthocephala: Echinorhynchidae) in schizothoracine fishes (Cyprinidae: Schizothoracinae) in Qinghai-Tibetan Plateau, China
Source: Parasit Vectors. 2020 Jul 20;13:357. doi: 10.1186/s13071-020-04224-w (PMC7372853; doi:10.1186/s13071-020-04224-w)
Supplement: Supplementary file 3 — Additional file 3: Table S3. Percentage nucleotide identity and numbers of nucleotide variations of ITS2 fragments from E. gymnocyprii populations. [file 13071_2020_4224_MOESM3_ESM.docx]

**Additional file 3: Table S3** Percentage nucleotide identity and numbers of nucleotide variations of ITS2 fragments from *E. gymnocyprii* populations

|  | QHL1 | QHL2 | MD1 | MD3 | MD8 | MD14 | DR5 | DR6 | DR7 | *E. gadi* |
| --- | --- | --- | --- | --- | --- | --- | --- | --- | --- | --- |
| QHL1 | - | 99.6 | 99.6 | 99.2 | 99.6 | 99.6 | 99.6 | 99.6 | 99.2 | 55.9 |
| QHL2 | 1 | - | 99.2 | 98.8 | 99.2 | 99.2 | 99.2 | 99.2 | 98.8 | 55.9 |
| MD1 | 1 | 2 | - | 98.8 | 99.2 | 99.2 | 99.2 | 99.2 | 98.8 | 55.5 |
| MD3 | 2 | 3 | 3 | - | 98.8 | 98.8 | 98.8 | 98.8 | 98.4 | 56.2 |
| MD8 | 1 | 2 | 2 | 3 | - | 99.2 | 99.2 | 99.2 | 98.8 | 56.2 |
| MD14 | 1 | 2 | 2 | 3 | 2 | - | 99.2 | 99.2 | 98.8 | 55.9 |
| DR5 | 1 | 2 | 2 | 3 | 2 | 2 | - | 99.2 | 98.8 | 56.2 |
| DR6 | 1 | 2 | 2 | 3 | 2 | 2 | 2 | - | 98.8 | 55.5 |
| DR7 | 2 | 3 | 3 | 4 | 3 | 3 | 3 | 3 | - | 55.1 |
| *E. gadi* | 112 | 112 | 113 | 111 | 111 | 112 | 111 | 113 | 114 | - |

Percentage nucleotide identity (above the diagonal), numbers of nucleotide variations (below the diagonal), ITS2 fragments of *E. gymnocyprii* populations (251 bp). The ITS2 fragments of QHL1, QHL3-5, MD2,MD4-7, MD9-13, MD15-18, DR2-4 and ZD1-5 were identical; therefore, data on only QHL1 is presented in this table. *E. gadi*, (EF107647.1) 234 bp.
