# Supplementary figures and images for: Prevalence and genetic diversity of Echinorhynchus gymnocyprii (Acanthocephala: Echinorhynchidae) in schizothoracine fishes (Cyprinidae: Schizothoracinae) in Qinghai-Tibetan Plateau, China
Source: Parasit Vectors. 2020 Jul 20;13:357. doi: 10.1186/s13071-020-04224-w (PMC7372853; doi:10.1186/s13071-020-04224-w)

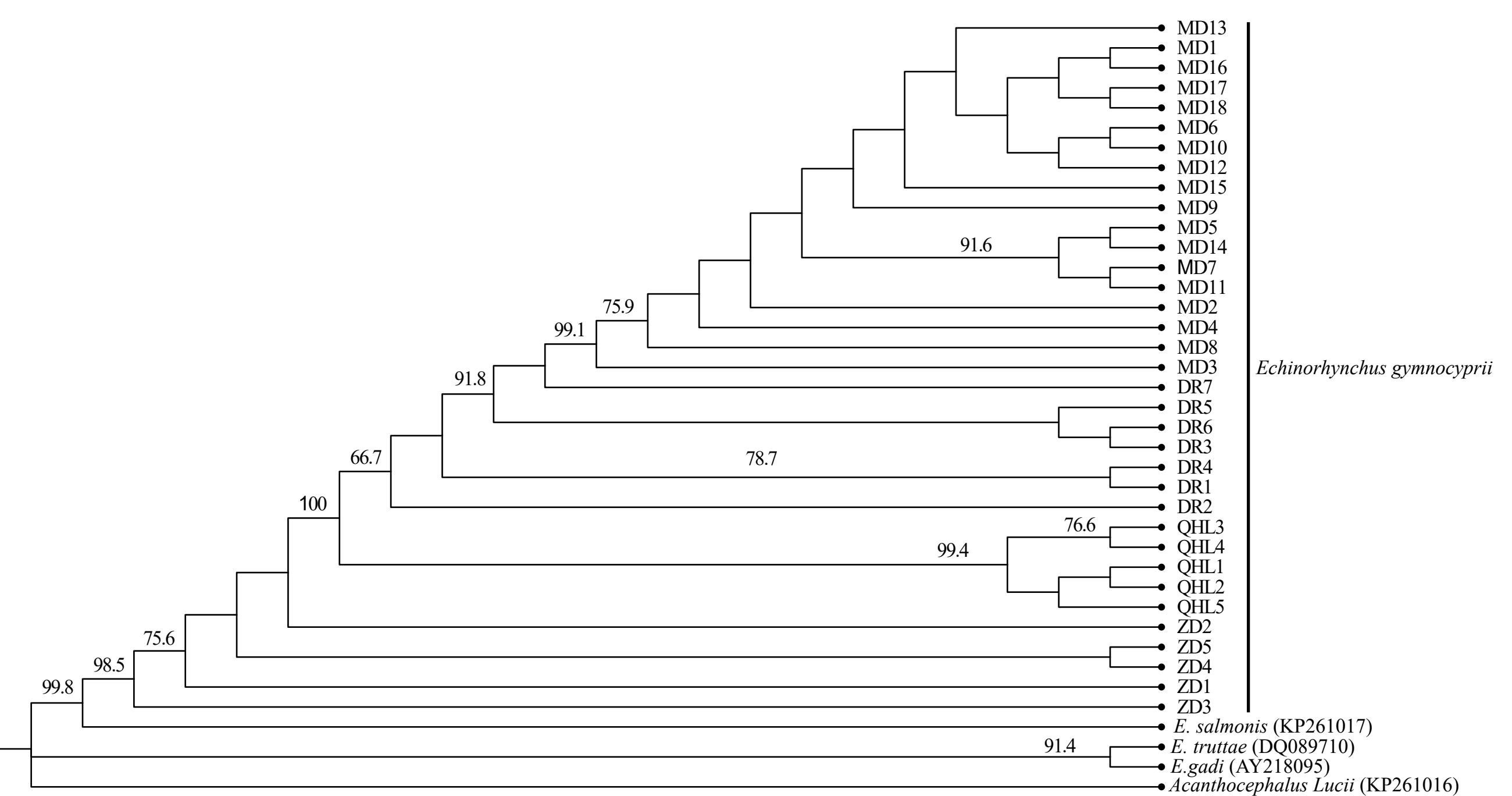

3.0

Supplement: Supplementary file 4 — Additional file 4: Figure S1. A phylogram form maximum likelihood (ML) analysis based on cox1 gene sequences depicting the relationships among the E. gymnocyprii sequences. [file 13071_2020_4224_MOESM4_ESM.pdf]
